# Uniform doping of graphene close to the Dirac point by polymer-assisted assembly of molecular dopants

**DOI:** 10.1038/s41467-018-06352-5

**Published:** 2018-09-27

**Authors:** Hans He, Kyung Ho Kim, Andrey Danilov, Domenico Montemurro, Liyang Yu, Yung Woo Park, Floriana Lombardi, Thilo Bauch, Kasper Moth-Poulsen, Tihomir Iakimov, Rositsa Yakimova, Per Malmberg, Christian Müller, Sergey Kubatkin, Samuel Lara-Avila

**Affiliations:** 1 Department of Microtechnology and Nanoscience, Chalmers University of Technology, 412 96 Gothenburg, Sweden; 2 Department of Physics and Astronomy, Seoul National University, Seoul, 08826 Korea; 3 Department of Chemistry and Chemical Engineering, Chalmers University of Technology, 41296 Göteborg, Sweden; 4 Institute of Applied Physics, Seoul National University, Seoul, 08826 Korea; 5 Department of Physics and Astronomy, University of Pennsylvania, Philadelphia, PA 19104 USA; 6 Department of Physics, Chemistry and Biology, Linkoping University, 581 83 Linköping, Sweden; 7 National Physical Laboratory, Hampton Road, Teddington, TW11 0LW UK

## Abstract

Tuning the charge carrier density of two-dimensional (2D) materials by incorporating dopants into the crystal lattice is a challenging task. An attractive alternative is the surface transfer doping by adsorption of molecules on 2D crystals, which can lead to ordered molecular arrays. However, such systems, demonstrated in ultra-high vacuum conditions (UHV), are often unstable in ambient conditions. Here we show that air-stable doping of epitaxial graphene on SiC—achieved by spin-coating deposition of 2,3,5,6-tetrafluoro-tetracyano-quino-dimethane (F4TCNQ) incorporated in poly(methyl-methacrylate)—proceeds via the spontaneous accumulation of dopants at the graphene-polymer interface and by the formation of a charge-transfer complex that yields low-disorder, charge-neutral, large-area graphene with carrier mobilities ~70 000 cm^2^ V^−1^ s^−1^ at cryogenic temperatures. The assembly of dopants on 2D materials assisted by a polymer matrix, demonstrated by spin-coating wafer-scale substrates in ambient conditions, opens up a scalable technological route toward expanding the functionality of 2D materials.

## Abstract (web summary)

Incorporating dopants in the graphene lattice to tune its electronic properties is a challenging task. Here, the authors report a strategy to dope epitaxial large-area graphene on SiC by means of spin-coating deposition of F4TCNQ polymers in ambient conditions.

## Introduction

Homogeneous doping of graphene down to low carrier densities enables the study of delicate and fascinating physics at the Dirac point. The prime experimental archetypes for the study of Dirac physics in solid-state devices are van der Waals (VdW) hetero-structures of graphene and hexagonal boron nitride (hBN). In these, extremely low carrier densities in graphene are attainable, especially when prepared on atomically flat, electrically conductive substrates that screen long-range potential fluctuations (i.e. disorder) in the graphene layer^1,2^. Typically microscopic in size, VdW hetero-structures are prone to size-induced mesoscopic effects that obscure novel physical effects expected in graphene at charge neutrality. Large-area graphene, uniformly doped to the Dirac point as in the case of VdW hetero-structures, is required to unveil the intimate details of, for instance, metal-insulator transitions^3^, interacting Dirac fermions^4^, and Zitterbewegung of electrons in graphene^5^. Atomically flat, large-scale epitaxial graphene on SiC (SiC/G) has demonstrated its potential for scalability in applications that demand extremely high-quality graphene over large areas, including quantum metrology^6,7^ and high-frequency electronics^8,9^. Yet, in practice, carrier density control of SiC/G remains a challenge because of high intrinsic n-doping (*n* > 10^13^ cm^−2^) due to interaction with the substrate, making it difficult to substantially tune its carrier concentration^10^. While numerous gating techniques have been applied to SiC/G to reduce its carrier density^8,9^, such approaches introduce carrier density fluctuations, which prevent further explorations of Dirac physics in this material.

Here we show a molecular approach to control the carrier density of epitaxial graphene homogeneously over macroscopic areas, yielding graphene with low carrier density (*n* < 10^10^ cm^−2^), low charge fluctuations (at the level of Δ*n* ≈ ± 6 × 10^9^ cm^−2^), and carrier mobilities up to 70 000 cm^2^ V^−1^ s^−1^ at low temperatures. So far, such electron transport properties have only been attained in microscopic single-crystal graphene flakes encapsulated by hBN^11^ or in suspended graphene^12^. The doping method described here was applied to samples over 5 × 5 mm^2^, resulting in epitaxial graphene devices that display quantum Hall effect at magnetic fields *B* < 1 T. These measurements indicate, as we elaborate below, a highly homogeneous spatial distribution of charge carriers on graphene. Chemical analysis reveals that air-stable molecular doping of graphene is achieved when organic molecules, embedded in a polymer matrix, diffuse through the matrix and spontaneously accumulate at the graphene surface due to formation of a charge-transfer complex. High-electron-affinity 2,3,5,6-tetrafluoro-tetracyano-quino-dimethane (F4TCNQ) molecules are mixed in a liquid solution with poly(methyl-methacrylate) (PMMA) and spin-coated at ambient conditions on SiC/G, with graphene being used both as the target substrate for molecular assembly and, simultaneously, as a charge sensor. The stability of the samples allows us to study the chemical composition as well as electronic transport properties of the F4TCNQ/graphene system. We expect this method to open up a scalable route toward expanding the properties and functionality of graphene and other two-dimensional (2D) materials well beyond doping control.

## Results

### Doping schemes

**Fig. 1:**
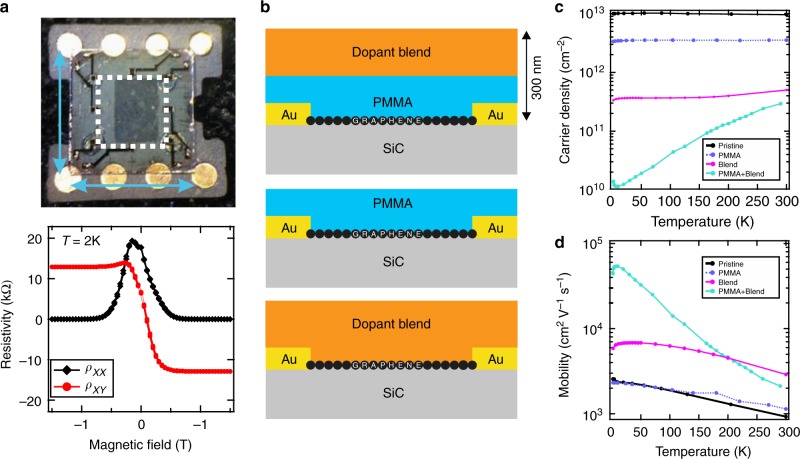
Magnetotransport characterization of chemically doped SiC/G. **a** Top: macroscopic graphene Hall bar device (white dotted outline, *W* = 5 mm × *L* = 5 mm). Bottom: low field magnetoresistance and fully developed quantum Hall effect indicating low charge disorder in chemically doped graphene, even over macroscopic areas. For this device, the measured carrier density *p* = 9 × 10^9^ cm^−2^ and mobility *μ* = 67 000 cm^2^ V^−1^ s^−1^. **b** The different encapsulation schemes with a polymer stack consisting of PMMA-F4TCNQ dopant blend, which comprises of F4TCNQ molecules in a PMMA matrix (F4TCNQ 7 wt%). The three schemes are dopant blend separated from graphene by a PMMA spacer (top), only PMMA layer (middle), and the dopant blend directly on the surface of graphene (bottom). **c** Carrier density as a function of temperature extracted from Hall measurements on small epitaxial graphene devices (*W* = 2–50 μm × *L* = 4–180 μm). **d** The corresponding Hall carrier mobility showing the highest *μ* ~ 55 000 cm^2^ V^−1^ s^−1^ at *T* = 10 K for sample prepared with PMMA spacer and dopant layer. The downturn in mobility at lower temperatures is due to quantum corrections to the Drude resistance. Carrier concentration *n* and mobilities *μ* were extracted from Hall measurements as *n* = 1/*eR*_H_ and *μ* = *R*_H_/*ρ*_*XX*_, with *e* the elementary charge, the Hall coefficient *R*_H_ = d*R*_*XY*_/d*B*, the longitudinal sheet resistance *ρ*_*XX*_ = *R*_*XX*_*W*/*L*, and *R*_*XY*_ the transversal resistance

The different chemical doping schemes of graphene devices are shown in Fig. 1b. Our devices include large (*W* = 5 mm × *L* = 5 mm) (Fig. 1a) and small (*W* = 2–100 μm × *L* = 10–180 μm) epitaxial graphene Hall bars fabricated by conventional electron beam lithography (see Methods section). When PMMA is used as a spacer between graphene and the molecular dopant layer, the carrier density decreases three orders of magnitude from its pristine value, *n* ≈ 1 × 10^13^ cm^−2^ to near charge neutrality, *n* ≈ 1 × 10^10^ cm^−2^ (Fig. 1c). Importantly, even at such low carrier densities the carrier mobility remains high, with the largest measured value exceeding *μ* ≈ 50 000 cm^2^ V^−1^ s^−1^ at *T* = 2 K. The effect of doping is homogeneous over millimeter scale and large samples display quantum Hall effect at magnetic fields *B* < 1 T (Fig. 1a), retaining their low carrier density over the course of 2 years, even under ambient conditions (Supplementary Fig. 1). To achieve this, a 200-nm-thick layer of the PMMA-F4TCNQ dopant blend is spin-coated onto a PMMA-protected sample, followed by thermal annealing above the PMMA glass transition temperature. The resulting carrier density could be fine-tuned by the total annealing time. For a concentration of 7% of F4TCNQ in PMMA by weight, charge-neutral graphene is achieved after annealing at *T* = 160 °C for 5 min. Shorter annealing times yield hole-doping and longer times yield electron-doping (Supplementary Fig. 1). Using the optimal time, we have consistently observed a decrease in electron density by three orders of magnitude together with a tenfold increase of carrier mobility at *T* = 2 K in more than 20 devices on 11 different chips (Supplementary Table 1 and Supplementary Fig. 2). Typical carrier concentrations in doped samples are of the order of 5 × 10^11^ cm^−2^ at room temperature. They decrease to values ∼1 × 10^10^ cm^−2^ at *T* = 2 K, due to freezing of thermally activated carriers^13^. The corresponding carrier mobilities are in the range of 30 000–50 000 cm^2^ V^−1^ s^−1^ (Fig. 1d). The PMMA spacer layer plays a crucial role in achieving high carrier mobilities. While both PMMA and the dopant blend act independently as moderate p-dopants when deposited directly on SiC/G, it is only when the PMMA spacer layer is added between graphene and the dopant blend that we observe the near to charge neutrality doping effect. Similarly, carrier mobilities exceed 10 000 cm^2^ V^−1^ s^−1^ only if the PMMA spacer and dopant blend operate in tandem (Supplementary Fig. 3).

### Chemical analysis of graphene-polymer hetero-structure

**Fig. 2:**
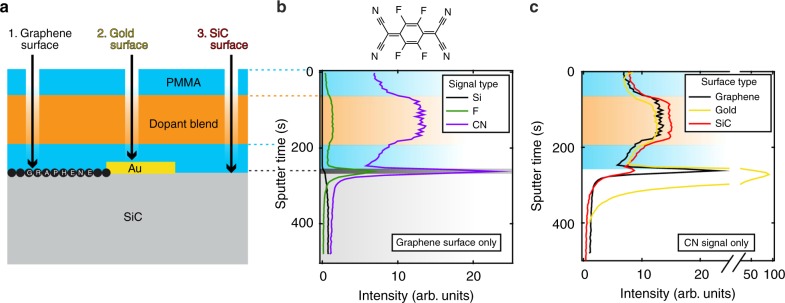
Chemical profiling of the polymer layers using ToF-SIMS to detect fingerprints of the F4TCNQ molecule (F and CN ions) as one probes deeper into the polymer stack. **a** Samples were prepared with PMMA spacer, dopant blend, and PMMA encapsulation layer. Three distinct spots on the substrate have been investigated: (1) graphene, (2) gold, and (3) SiC surfaces. **b** When analyzing the polymer layers on spot 1, above the graphene surface, the ion intensity for F and CN ions (top inset shows a schematic representation of a F4TCNQ molecule) vs. sputter time reveals significant accumulation of F4TCNQ at the graphene/PMMA interface, as well as the spatial distribution of F4TCNQ molecules through the spacer and encapsulation PMMA layers. The onset of the silicon signal (Si) is the marker, which indicates that the SiC substrate has been reached. The shaded regions denote the extent of each layer, from top PMMA layer down to SiC substrate. **c** Here we focus only on the CN signal measured at all three different spots. This analysis shows accumulation of F4TCNQ on the conductive surfaces of graphene and gold, but virtually no accumulation on SiC

We demonstrate that the doping of graphene is the result of F4TCNQ molecules diffusing through the PMMA layer and accumulating at the graphene surface. Figure 2a, b shows the chemical depth profile of the polymer stack, obtained by using time-of-flight secondary ion mass spectrometry (ToF-SIMS), revealing both diffusion of F4TCNQ through the PMMA spacer and the accumulation of molecules at the graphene surface. From this we estimate the diffusion coefficient of F4TCNQ through PMMA to be of the order of 10^14^ cm^2^ s^−1^ and by integrating the areas under the ion current intensity curves, we estimate the density of F4TCNQ molecules near the graphene surface to be ~4.6 × 10^14^ cm^−2^ (Supplementary Fig. 4, Supplementary Note 1). Graphene and metallic surfaces promote the accumulation of F4TCNQ, while there are virtually no dopant molecules at the polymer/SiC interface. The surface density of F4TCNQ is roughly 50% greater on graphene (and sixfold higher on gold) compared to that in the dopant blend layer (Fig. 2c). We attribute the accumulation of F4TCNQ on the graphene surface and the measured p-doping effect to the formation of a charge transfer complex, with partially charged F4TCNQ remaining at the graphene interface to preserve overall charge neutrality. F4TCNQ is known to be mobile in thin polymer films^14,15^, with its diffusion depending on polarity and glass transition temperature of the polymer. When using an inert PMMA as a host matrix, F4TCNQ remains neutral both in the doping layer and as it diffuses through PMMA spacer layer^16^. The formation of a charge transfer complex takes place only when encountering an electron donor, such as graphene. Once charged, the F4TCNQ anion is bound to graphene, stabilized by Coulomb interaction^17^.

### Low charge disorder in SiC/G doped close to Dirac point

**Fig. 3:**
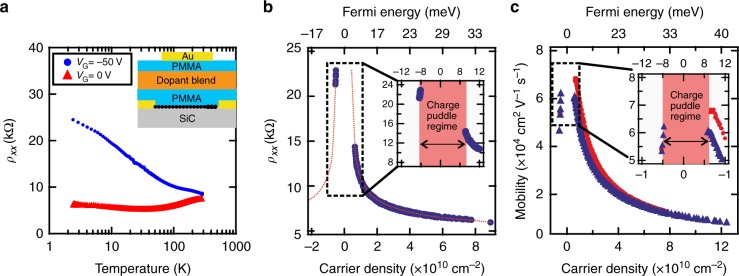
Electrostatic gating of chemically doped graphene (30 μm × 180 μm Hall bar). **a** Temperature dependence of longitudinal resistance for graphene in the metallic limit (red) and gated to Dirac point (blue). (Inset) Schematic representation of a chemically doped graphene device with a metallic gate (the topmost Au layer). **b** *ρ*_*XX*_ vs. carrier concentration shows the characteristic Dirac peak around the charge neutrality point (red dotted line serves as a guide to the eye). The Dirac point is crossed below Vg = −40 V, and the device has well-defined carrier densities within ±6 × 10^9^ cm^−2^ at *T* = 2 K, which corresponds to a Fermi energy of ±9 meV. **c** Corresponding charge carrier mobilities, with values up to 70 000 cm^2^ V^−1^ s^−1^. Each point in **b** and **c** represents data of magnetic field scans where *R*_*XY*_ is linear in the low magnetic field limit and the device shows fully developed quantum Hall effect at high magnetic fields (*ρ*_*XX*_ = 0 and exactly quantized *R*_*XY*_ plateaus). The gap in data around zero carrier concentration corresponds to omitted data points where graphene is in the charge puddle regime. For comparison, in **c**, red circles and blue triangles correspond to Hall measurements from two different Hall probe pairs on the same device. The gate voltage was applied at cryogenic temperatures; the measured leakage current did not exceed 50 pA, with the bias current on graphene of 100 nA

We investigated further electron transport details of the F4TCNQ-graphene charge-transfer complex system by introducing a top electrostatic gate (Fig. 3a, inset) on top of a 30 μm × 180 μm Hall bar. The top gate enables additional fine tuning of the carrier concentration within Δ*n* ~ 5 × 10^11^ cm^−2^ using gate voltages *V*_G_ = −100 to +200 V. At *V*_G_ = 0 V the carrier density is *n* = 5 × 10^11^ cm^−2^ at room temperature and graphene is in the metallic limit. In this case, *ρ*_*XX*_(*T*, *V*_G_ = 0 V) decreases linearly with temperature from its room temperature value, due to suppression of acoustic phonon scattering. Quantum corrections to resistance result in a log(*T*) dependence below the Bloch-Grüneisen temperature ${{T}}_{{\mathrm{BG}}} = 2v_{{\mathrm{ph}}}{{E}}_{\mathrm{F}}/\left( {k_{\mathrm{B}}v_{\mathrm{F}}} \right) = 2v_{{\mathrm{ph}}}\hbar v_{\mathrm{F}}\sqrt {\pi n} /\left( {k_{\mathrm{B}}v_{\mathrm{F}}} \right) \approx 38\,{\mathrm{K}}$, with the phonon velocity *ν*_ph_ = 2 × 10^4^ m s^−1^ (${{E}}_{\mathrm{F}} = \hbar v_{\mathrm{F}}\sqrt {\pi n}$ the Fermi level of graphene, *ħ* the reduced Planck’s constant, *k*_B_ the Boltzmann constant, and *ѵ*_F_ = 10^6^ m s^−1^ the Fermi velocity in graphene^18^). In contrast, when graphene is gated to the Dirac point, the sheet resistance *ρ*_*XX*_(*T*, Vg = −50 V) monotonically increases as the temperature drops, though remains finite with no indication of a transport gap in the current voltage characteristics down to *T* = 2 K (Fig. 3a).

To characterize the magnitude of carrier density fluctuations (i.e. how precisely one can approach the Dirac point) we conducted low-temperature magnetotransport measurements on chemically and electrostatically gated devices and found these fluctuations to be on the level of Δ*n* ~ ± 6 × 10^9^ cm^−2^ (Δ*E*_F_ ~ ± 9 meV). Figure 3b shows longitudinal resistance vs. carrier concentration in which every data point, extracted from individual measurements of *ρ*_*XX*_(*B*), *R*_*XY*_(*B*) at a fixed gate voltage, corresponds to devices behaving as a system with a single electronic band and spatially homogenous carrier density^19–23^. That is, data points in Fig. 3b fulfill simultaneously the criteria of linear *R*_*XY*_(*B*) at low fields^19–22^, and fully developed half-integer quantum Hall Effect at high fields^23^, i.e. *ρ*_*XX*_(*B*) = 0 Ω and strictly quantized *R*_*XY*_ plateau over the entire available range of magnetic field (Supplementary Fig. 5). The gap in data around zero carrier concentration thus corresponds to data points where graphene is in the charge puddle regime. Under quantizing conditions, the residual disorder in the sample causes non-zero and oscillatory *ρ*_*XX*_(*B*) once the magnetic length approaches the average charge puddle size^23,24^. In our samples with low carrier density concentration ~10^10^ cm^−2^ we have observed no deviation from *ρ*_*XX*_(*B*) = 0 to the largest magnetic field available in our setup (*B* = 14 T) (Supplementary Fig. 5). Thus, we establish an upper limit for the puddle size of about $l_{\mathrm{B}} = \sqrt {\hbar /{{eB}}} \approx 7\,{\mathrm{nm}}$. The charge puddle magnitude is directly connected to disorder introduced by, e.g., topography or inhomogeneous doping^25^. The small magnitude of charge puddles measured in our devices indicate that SiC/G doped with F4TCNQ molecules is homogenous also at the microscopic scale, with carrier density fluctuations comparable to those in high-quality, hBN-encapsulated single-crystal graphene flakes obtained by exfoliation^11^ or by chemical vapor deposition growth^26^ (Supplementary Fig. 6).

**Fig. 4:**
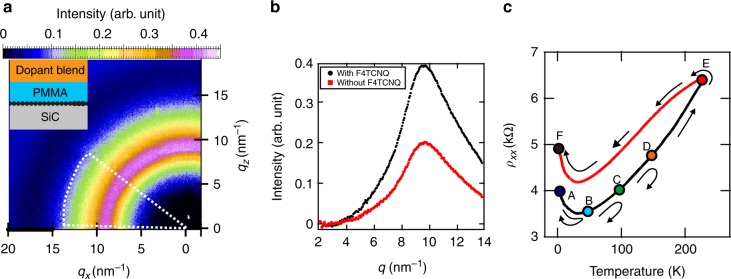
F4TCNQ in PMMA matrix. **a** Grazing-incidence wide-angle x-ray scattering (GIWAXs) measurements taken on SiC/G with dopant blend and PMMA spacer (inset) (ambient condition, room temperature, 0.15° incident angle). The diffraction ring with radius *q* = 9.6 nm^−1^ from PMMA and the absence of F4TCNQ diffraction spots suggests a good molecular solubility in the polymer matrix. The color bar shows the normalized intensity, after a constant diffuse scattering background has been subtracted. The white dotted region denoted over which azimuthal angles the intensity profile in **b** has been averaged. **b** The reference sample, without F4TCNQ in the dopant blend displays a clear peak diffraction ring with radius *q* = 9.6 nm^−1^; the addition of F4TCNQ molecules enhances the signal twofold. **c** Stability of doping at low temperature in a device that has been cooled down from room temperature. Starting at *T* = 300 K we applied a gate voltage *V*_G_ = +50 V during cool down. Once we reached *T* = 2 K, the gate terminal is set to *V*_G_ = 0 V and sample sheet resistance acquires a value of *ρ*_*XX*_ = 4 kΩ (point A). Thermal excursions to *T* = 50 K (B), 100 K (C), and 150 K (D) result in reversible *ρ*_*XX*_(*T*) along the black curve. Once temperature exceeds *T* = 150 K (E), *ρ*_*XX*_(*T*) irreversibly changes to a higher resistive value (red curve), and on cooling down back to *T* = 2 K, the sample resistance takes a value of *ρ*_*XX*_ = 5 kΩ (F). In the absence of the gate voltage, the sample resistance remains in the higher resistance *ρ*_*XX*_(*T*) branch

It is remarkable that epitaxial graphene displays such low disorder even at extreme dopant coverage, being decorated with a dense layer of molecules of about 3–4 molecules per nm^2^ (*c* ≈ 3 × 10^14^ cm^−2^, comparable to the molecular coverage of *c* ≈ 4.6 × 10^14^ cm^−2^ from SIMS). We estimated the molecular density at the graphene surface from the shift in carrier density measured in doped graphene with respect to its pristine concentration (Δ*n* ≈ 1 × 10^13^ cm^−2^), and assuming that 0.3 electrons are withdrawn from epitaxial graphene per molecule^27,28^ with 1/10 gate efficiency^10^. The homogeneity in doping is in part enabled by the high degree of F4TCNQ dispersion inside the PMMA matrix, shown by room temperature grazing-incidence wide-angle X-ray scattering (GIWAXs). The diffractogram in Fig. 4a, b reveals a broad amorphous halo with a distinct diffraction peak at *q* = 9.6 nm^−1^ from PMMA (Supplementary Note 2). The absence of diffraction spots from F4TCNQ implies the lack of molecular aggregation (i.e. crystallites) inside the matrix. Given the size of the F4TCNQ molecule, we propose that at this packing density a feasible molecular orientation of F4TCNQ is close to that of molecules standing up on the graphene surface^28^. Yet, we do not rule out molecular re-orientation or thermally induced redistribution of charges in the dopant layer under the effect of electric field, even at low temperatures. Such charge redistribution in the dopants in close vicinity to graphene may be responsible for screening charge inhomogeneities that facilitate highly uniform doping^1^. Thermally activated motion of charges in the dopant layer is a plausible source of the hysteresis in *ρ*_*XX*_(*T*) when devices are subjected to thermal excursion from *T* = 2 to 230 K (Fig. 4c). This is also suggested by more accurate resistance measurements, which reveal that charges in the dopant layer are mobile down to *T* ≈ 113 K (Supplementary Fig. 7), in notable coincidence with the energy scale of the measured charge inhomogeneity in doped epitaxial graphene (Δ*E*_F_ ~ ± 9 meV). The charge homogeneity of the samples is thus determined by the temperature at which the screening charges freeze.

## Discussion

A possible explanation for the observed low disorder and high charge carrier mobility of SiC/G at large molecular coverage is a high degree of spatial correlation between adsorbed F4TCNQ and impurities present on bare graphene. Together with the low charge carrier density fluctuations, increasing impurity (e.g. F4TCNQ) density can lead to suppression of charge scattering in graphene if there is sufficient inter-impurity correlation present in the system^29,30^ (Supplementary Fig. 8). The actual organization and conformation of charged molecules on graphene is the result of delicate balance between molecule-graphene interactions as well as the intermolecular many-body dispersion forces^31^ in the presence of polymer. While the exact arrangement of molecules on graphene is difficult to probe through the thick polymer layers with surface science techniques, the graphene layer itself utilized as detector allows gaining an insight of such molecular reorganization at the polymer-graphene interface.

In summary, we presented a method of guiding the assembly of molecular dopants onto the surface of graphene by using an organic polymer matrix. With the doping stability observed in our samples, the tuneability of molecular coverage, and given the vast catalog of polymers and organic/organo-metallic molecules, we expect this method to open up a scalable route toward expanding the properties and functionality of graphene and other 2D materials well beyond doping control. Moreover, the presented analysis of chemical and electron transport properties of doped graphene sheds light on the complex processes that molecular dopants undergo when embedded in polymers. This is relevant to the understanding of performance, materials, and interfaces in organic electronic devices, especially when combined with 2D materials. This method can be explored in the future to create and study electron transport properties of novel 2D systems of ordered molecular arrays templated by 2D crystals^32–35^.

## Methods

### Sample fabrication

Monolayer SiC/G was grown on the Si-face of SiC using thermal decomposition of 7 mm × 7 mm 4H(0001)-SiC substrates. The samples were grown in argon atmosphere of 800 mbar, and kept at a temperature of around 1700 °C for 5 min^36^. Optical microscopy^37^ revealed a typical surface coverage of >95% monolayer graphene.

Epitaxial graphene Hall bars were fabricated using electron beam lithography described in detail elsewhere^38^. The electrical contacts were fabricated using physical vapor deposition of Ti and Au, 5 and 80 nm thick, respectively. After fabrication samples were cleaned using isopropyl alcohol, acetone, and dried using nitrogen gas. For pristine SiC/G (shown in Fig. 1b) a hBN flake was exfoliated and transferred to pristine SiC/G using a polydimethylsiloxane stamp. To clean the interface the sample was annealed in argon 800 mbar at 755 °C, after which standard lithography was performed.

The dopant blend consisted of a mixture of F4TCNQ (Sigma-Aldrich) and PMMA (Microlithography Chemicals Corp., molecular weight 950 000). A unit of 25 mg of dry F4TCNQ powder was mixed with 3 ml anisole solvent. A volume of 0.5 ml of this solution was then mixed with 1 ml PMMA A6 (6% PMMA by weight in anisole). The PMMA spacer layer and encapsulation layers used either PMMA A4 (5% by weight in anisole) or PMMA-based copolymer (MMA (8.5) MAA) EL6 (6% by weight in ethyl lactate solvent). All polymer layers were deposited on graphene using spin-coating at 6000 rpm for 1 min. After each spin-coating step a 5 min baking step on a hotplate set at 160 °C follows. The dopant blend is an exception, since the baking time dictates the final doping level.

The electrostatic top gate was fabricated using a shadow mask, and physical vapor deposition of Au in order to avoid disruption of the chemically doped sample. In order to preserve the integrity of the doping strength of graphene, additional layers of dopant blend and PMMA encapsulation layers were deposited before fabrication of the gate. The samples with the full stack of five polymer layers are the most resilient to drift in carrier concentrations in ambient conditions.

### Electrical measurements

Standard electrical characterization was performed in a liquid ^4^He gas flow cryostat, which allowed for temperatures down to 2 K and magnetic fields up to 14 T. All reported values of charge carrier mobility and charge carrier concentration were extracted from four-probe Hall and quantum Hall measurements. The standard measurement setup used current biased samples at maximum of 100 nA DC (Keithley 6221 DC and AC current source, Agilent 34420 A nanovolt meter), in this range samples showed linear *IV* characteristics down to cryogenic temperatures. The electrostatic top gate was DC biased with voltages ranging from −100 to +200 V, where leakage current was <0.05 nA.

### Secondary ion mass spectrometry

ToF-SIMS analysis was performed using a TOF.SIMS 5 instrument (ION-TOF GmbH, Münster, Germany). The instrument is equipped with a 25 keV Bi_3_^+^ cluster ion gun as the primary ion source and a 10 keV C_60_^+^ ion source for sputtering and etching. The samples were analyzed using a pulsed primary ion beam (Bi_3_^++^, 0.34 pA at 50 keV) with a focus of approximately 2 μm at a field of view of 150 µm × 150 µm. The mass resolution using this setup was at least *M*/Δ*M* = 5000 full width at half maximum at *m*/*z* 500. All spectra were acquired and processed with the Surface Lab software (version 6.4, ION-TOF GmbH). Depth profile analysis was performed using a C_60_^++^ beam at 20 keV with a current of 0.2 nA in a non-interlaced mode with 1 s of analysis, 1 s of sputtering, and a pause of 1 s for each sputter cycle at 350 µm × 350 µm. The maximum ion dose density of Bi_3_^++^ was kept between 4 × 10^12^ and 7 × 10^12^ cm^−2^ over the whole depth profiling experiment, while the ion dose for C_60_^++^ ranged from 2 × 10^14^ to 4 × 10^14^ ions per cm^2^. Low-energy electrons were used for charge compensation during analysis.

### Grazing-incidence wide-angle X-ray scattering

Data were obtained at the D1-beam line at the Cornell High Energy Synchrotron Source at Cornell University. The measurements used a positron beam with synchrotron radiation of a wavelength of 1.162 Å, Pilatus 200K detector with pixel size of 172 μm × 172 μm and sample to detector distance of 177.2 mm. Measurements were done in ambient conditions at room temperature. The exposure time for each individual measurement was 20 s.

## Electronic supplementary material

Supplementary Information

Peer Review File

## Data Availability

The authors declare that the main data supporting the findings of this study are available within the article and its Supplementary Information files. Extra data are available from the corresponding author upon request.
