# Supplementary material for: Uniform doping of graphene close to the Dirac point by polymer-assisted assembly of molecular dopants
Source: Nat Commun. 2018 Sep 27;9:3956. doi: 10.1038/s41467-018-06352-5 (PMC6160407; doi:10.1038/s41467-018-06352-5)
Supplement: Supplementary file 1 — Supplementary Information [file 41467_2018_6352_MOESM1_ESM.pdf]

Supplementary information for “Uniform doping of graphene close to the Dirac point by polymer-assisted assembly of molecular dopants”

Hans He[1], Kyung Ho Kim[1,2], Andrey Danilov[1], Domenico Montemurro[1], Liyang Yu[3], Yung Woo Park[2,4,5], Floriana Lombardi[1], Thilo Bauch[1], Kasper Moth-Poulsen[3] , Tihomir Iakimov[6], Rositsa Yakimova[6], Per Malmberg[3], Christian Müller[3], Sergey Kubatkin[1] & Samuel Lara-Avila*[1,7]

1. Department of Microtechnology and Nanoscience, Chalmers University of Technology, SE-412 96, Gothenburg, Sweden

2 Department of Physics and Astronomy, Seoul National University, Seoul, 08826, Korea

3 Department of Chemistry and Chemical Engineering, Chalmers University of Technology, 41296 Göteborg, Sweden

4 Institute of Applied Physics, Seoul National University, Seoul, 08826, Korea

5 Department of Physics and Astronomy, University of Pennsylvania, Philadelphia, PA, 19104, USA

6 Department of Physics, Chemistry and Biology, Linkoping University, S-581 83, Linköping, Sweden

7 National Physical Laboratory, Hampton Road, Teddington, TW11 0LW, UK

***Correspondence and requests for materials should be addressed to S.L.A. email: samuel.lara@chalmers.se**

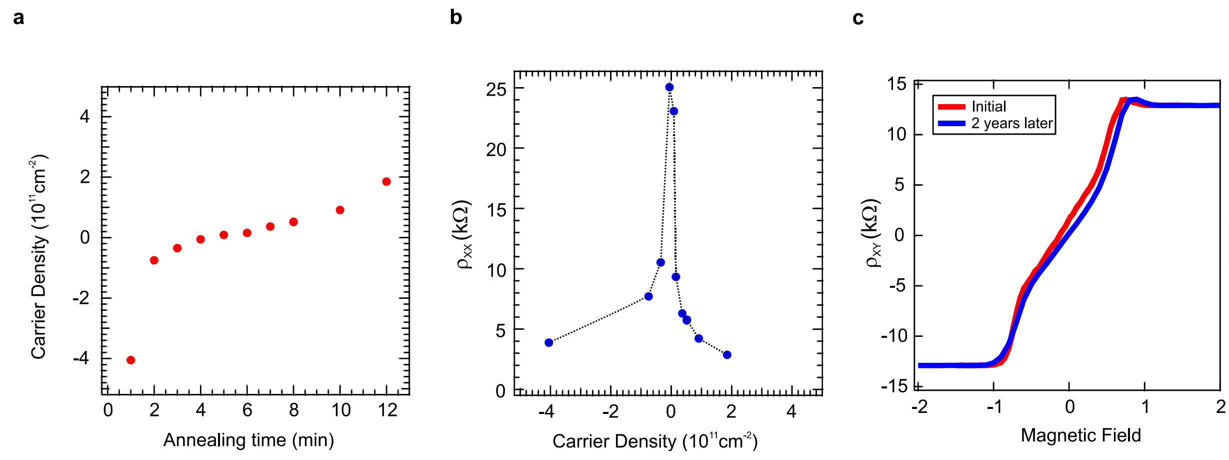

Supplementary Figure 1- Doping technique details and doping stability

(**a**) The standard combination of polymers consists of a spin coated spacer layer of PMMA (~100 nm) and then a spin coated dopant blend (~170 nm) on top. After each spin coating step the sample is annealed at 160 ˚C. The crucial part which determines the final doping level of samples is the baking of the dopant blend layer. The standard 5 min baking time of the dopant blend leaves SiC/G close to charge neutrality $n \sim 10^{10}$ cm^{-2}. All data are extracted from Hall/quantum Hall measurements and we also observe that the amount of charge disorder in samples, manifesting itself as non-linear low-field R_{XY} and non-zero R_{XX} under quantizing conditions, tends to decrease with increased baking time. (**b**) ρ_{XX} taken from the same data as (a) plotted versus carrier density reveals the typical Dirac peak. (**c**) Long-term stability for low n-doped samples, demonstrated by quantum Hall measurements taken two years apart. The samples which are most stable over time consist of two bi layers of PMMA encapsulation and dopant blend. Note that the annealing curve is different from the one in (a) for this configuration. The stability of the doping was investigated by re-measuring two separate samples on the same chip after roughly two years, during which they were kept under ambient conditions or in nitrogen-ventilated cabinet. It was found that the samples retained their low doping level, increasing from $n \sim 5.4 \times 10^{10}$ cm^{-2} to $n \sim 6.2 \times 10^{10}$ cm^{-2}, and show fully developed quantum hall plateaus below 2 T. From this we estimate that the degradation of the doping effect of the F4TCNQ molecules is less than 1%, relative to an initial doping strength of $\sim 10^{13}$ cm^{-2}.

Dev. No.	Chip ID	Wafer	Hall Bar ID	Area (μm^2)	|n| (cm^{-2})	u (cm^{2}V^{-1}s^{-1})
**1**	G287	Cree	LHB	5000 x 5000	9.10E+10	6.70E+04
**2**	G488	Cree	HB	10 x 10	5.50E+10	3.80E+04
**3**			HB	10 x 10	5.20E+10	4.00E+04
**4**	G782	Norstel#1	B4	30 x 180	5.80E+10	2.00E+04
**5**				30 x 180	7.90E+10	1.40E+04
**6**			E2	30 x 180	8.10E+09	5.10E+04
**7**			B1	30 x 180	9.60E+09	4.50E+04
**8**				30 x 180	8.90E+09	4.90E+04
**9**			A3	30 x 180	2.00E+10	3.80E+04
**10**				30 x 180	2.70E+10	2.90E+04
**11**	G783	Norstel #1	E1	30 x 180	4.70E+10	2.30E+04
**12**				30 x 180	7.10E+10	1.50E+04
**13**	G792	Norstel #1	A2	18 x 90	9.10E+09	4.50E+04
**14**			C2	18 x 90	6.00E+09	8.20E+04
**15**				18 x 90	6.50E+09	7.60E+04
**16**			B2	36 x 144	1.50E+10	4.80E+04
**17**				36 x 144	2.40E+10	3.00E+04
**18**			A3	36 x 144	1.40E+10	4.30E+04
**19**				36 x 144	9.60E+09	6.30E+04
**20**			A1	36 x 144	8.60E+09	5.80E+04
**21**				36 x 144	9.10E+09	5.50E+04
**22**			B4	36 x 144	1.80E+10	4.60E+04
**23**			C3	36 x 144	6.40E+10	2.30E+04
**24**	G793	Norstel #1	A1	2 x 4	4.00E+10	2.00E+04
**25**	G804	Norstel #2	A2	36 x 144	5.00E+10	2.70E+04
**26**			A3	36 x 144	9.00E+10	1.50E+04
**27**	G938	Norstel #1	LHB	5000 x 5000	1.87E+10	2.10E+04
**28**	G979	Norstel #2	A1	36 x 144	1.50E+10	4.50E+04
**29**	G1029	Norstel #3	MM	100 x 180	1.50E+10	3.00E+04
**30**			MR	100 x 180	2.80E+10	2.50E+04
**31**			ML	100 x 180	7.00E+10	1.40E+04
**32**	G1043	Cree	MHB	100 x 180	1.50E+10	4.10E+04

Supplementary Table 1 - Summary of measured SiC/G samples doped with F4TCNQ

A summary of all measured devices doped using F4TCNQ dopant blend and PMMA spacer layer. It is a comprehensive list of all of the measured devices and their area, charge carrier concentration and mobility. Note that each chip contains multiple Hall bars. Carrier concentration and mobility were extracted from quantum Hall measurements taken at T=2 K. We have tested the combination of PMMA spacer and dopant blend on 24 Hall bars spread over 11 separate 7 mm x 7 mm chips. The chips are diced from 3 inch SiC wafers (four different wafers from two suppliers, Cree Inc. and Norstel AB) and graphene was grown on each chip individually. The device geometry is a rectangular Hall bar, with dimensions ranging from small 2 μm x 4 μm to macroscopic 5000 μm x 5000 μm. Since values for carrier density n fulfill simultaneously the criteria of linear $R_{XY}(B)$ at low fields, and fully developed half-integer Quantum Hall Effect at high fields, the carrier density values in the table provide an upper boundary for disorder-induced charge density fluctuations. The variability might arise not only from the doping process itself, but also from as-grown epitaxial graphene quality (e.g. bilayer inclusions which can affect things on the device level).

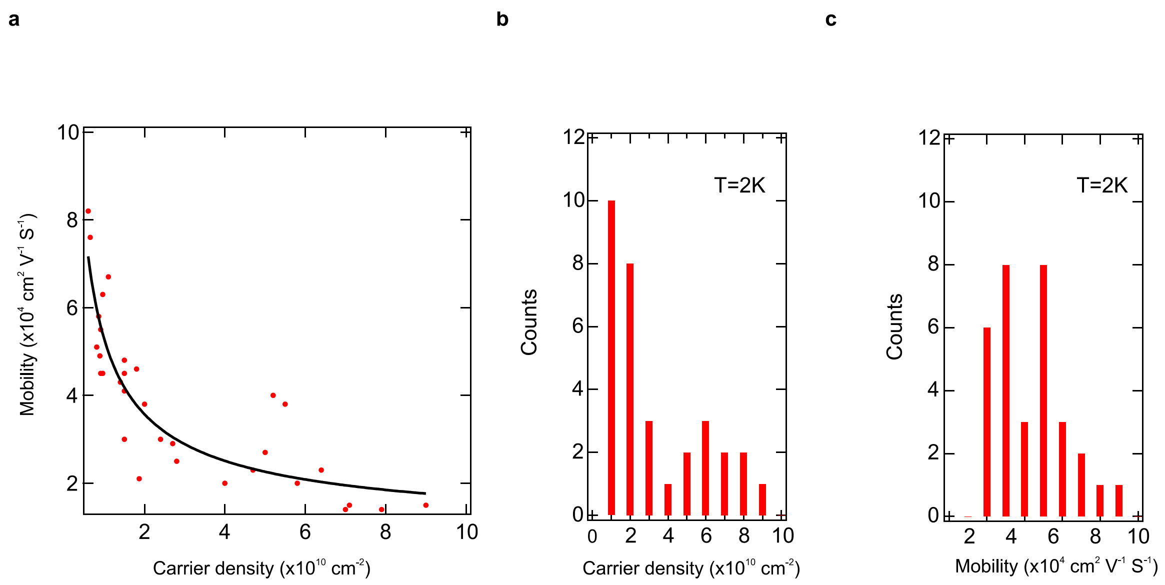

Supplementary Figure 2 - Statistics from Supplementary Table 1

All data are taken from Supplementary Table 1. (**a**) Carrier concentration versus mobility. The solid black line is a guide to the eye going as $\mu \propto 1/n$. (**b**) Histogram showing the spread of carrier concentration across all devices. The final doping level of SiC/G after spin coating and baking the dopant blend for 5 minutes, with a PMMA spacer layer, yields an average carrier concentration of $n_{avg}=3x10^{10} \pm 2.5x10^{10}$ cm^{-2}. (**c**) Histogram showing the spread of carrier mobility across all devices.

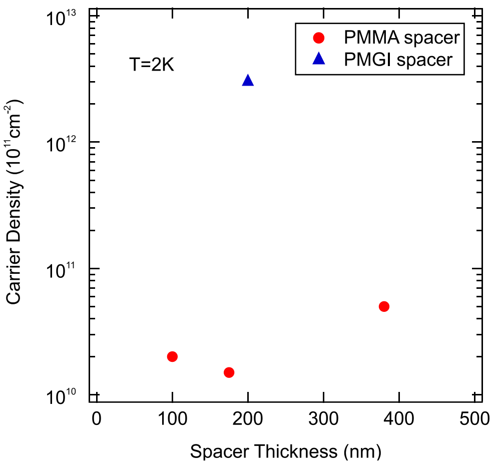

Supplementary Figure 3 – Spacer layer thickness

Different polymers and thicknesses have been tried as the spacer layer which separates graphene from the dopant blend containing F4TCNQ. No discernable difference was observed between PMMA and copolymer (MMA(8.5)MAA) spacer layers, and both resulted in close to charge neutral graphene for 5 min annealing time at 160 ˚C of the dopant blend. Furthermore, for PMMA-based spacer layer there is no clear correlation between spacer thicknesses (100-380 nm) and final doping on samples. Note that in the limit of thin spacer layer, e.g. only dopant blend, the final carrier concentration is high as seen in Fig. 1 in the main text. Additionally, we observe that polydimethylglutarimide-based spacer (LOR and PMGI resists, by MicroChem) hinders the doping effect of F4TCNQ molecules. Red dots indicate measured carrier concentration for graphene using PMMA spacer layer with dopant blend on top, baked for 5 min at 160 ˚C. Samples can still reach low carrier concentrations even with varying spacer thickness between 100-380 nm. The blue dot shows that for samples using PMGI spacer baked for 5 min at 160 ˚C. In this case the dopant blend has no effect of the carrier concentration, and samples remain highly n-doped.

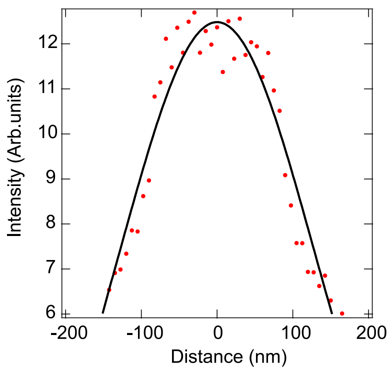

Supplementary Figure 4 – Diffusion of F4TCNQ in PMMA

Solving Fick's laws of diffusion for the case of instantaneous limited source diffusion with an extended source[1], yields the spatial change of surface concentration according to $c(x,t) = \frac{1}{2}c_0\left\{\operatorname{erf}\frac{h-x}{2\sqrt{Dt}} + \operatorname{erf}\frac{h-x}{2\sqrt{Dt}}\right\}$. Here c_0 is the initial surface concentration in the dopant blend, the source extends from –h to h, x the distance from the center of the source, t is time and D is the diffusion coefficient. Fitting this equation to SIMS data, which is a measurement of surface concentration, we estimated diffusion coefficient D~10^{-14} cm^2 s^{-1} for F4TCNQ when the PMMA matrix is baked for 6,000 seconds at 160 ˚C. The sputtering time of the SIMS has been roughly translated to distance x to match the known resist thicknesses (assuming a constant sputter rate). The fit quality is acceptable, though the fit is not perfect due to the assumptions of an instantaneous source, constant diffusion coefficient, and ignoring accumulation of dopants at the graphene surface. Limited source diffusion fit (solid black line) to SIMS data (red dots), taking into consideration the region above and below the dopant blend layer. The accumulation layer near graphene has been omitted. The SIMS data has been rescaled from sputter seconds to distance (nm) according to the known resist thickness and a constant background has been subtracted. The center (x=0 nm) indicates the center of the dopant blend layer, negative x goes towards the top of the chip and positive x goes towards SiC/G.

Supplementary Note 1 – SIMS data

From the SIMS data (Fig. 2) we find, by taking the ratio of the area under the intensity curve for the accumulation layer and the whole curve, that ~15% of the F4TCNQ molecules in the dopant blend accumulated near graphene. Since the concentration of F4TCNQ in PMMA is known to be 7% wt. %, and assuming that the dopant blend, after baking, is a solid F4TCNQ/PMMA slab of 7 mm x 7 mm x 180 nm, the density of molecules near the graphene can be estimated to be ~4.6x10^{14} molecules/cm^2. Given the polarity of PMMA and the thermal annealing step of our process above the glass transition temperature of the polymer, a conservative estimate for the lower bound of the flux of F4TCNQ at the substrate surface is $j = D\frac{\Delta c}{\Delta d} = 1.5x10^{-13}$ mol cm^{-2} s^{-1}, which means that 4.6x10^{14} molecules cm^{-2} of F4TCNQ reaches the graphene at the spacer/substrate interface in 5,000 seconds, which corresponds well to the 6,000 seconds baking time at 160 ˚C. Here we have used D ~ 10^{-14} cm^2 s^{-1} extracted from fit to SIMS data, Δc=2.69x10^{-4} mol cm^{-3} the initial F4TCNQ concentration gradient between the doping and bottom spacer layer, and Δd=180 nm is the distance from the center of the dopant layer to graphene (including 100 nm PMMA spacer). In the above calculations we have assumed that the density of F4TCNQ is ρ~1.4 g cm^{-3} and molar mass is M~276 g mol^{-1}.

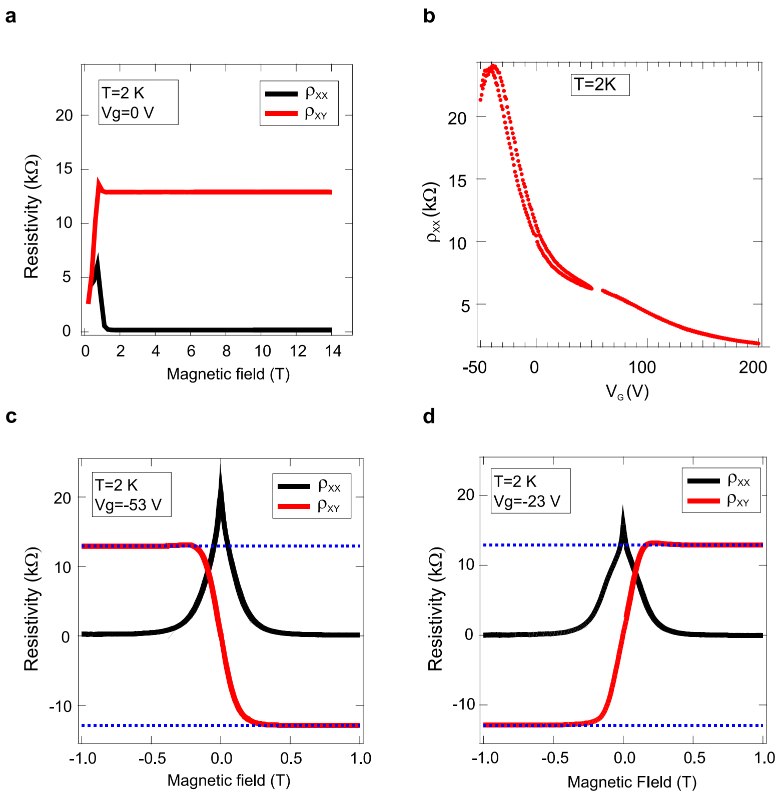

Supplementary Figure 5 - Quantum Hall effect close to Dirac Point

(**a**) Example of high-field quantum Hall effect for chemically doped SiC/G sample with $n=3x10^{10}$ cm^{-2}. Fully developed quantum Hall effect occurs below 2 T, and maintained up to maximum tested field of 14 T. (b)-(d) show measurements performed on chemically doped SiC/G with an additional electrostatic top gate. (**b**) Gate voltage sweep through the Dirac point, with the peak occurring close to Vg=-40 V. (**c**) Quantum hall measurements performed at Vg=-53. The sample shows full quantization below 1 T in p-doped regime $p=5.6x10^{9}$ cm^{-2} and mobility μ= 52,000 cm^{2} V^{-1} s^{-1} . The blue dotted line indicates one resistance quantum $h/2e^{2}$ (**d**) At V_G=-23 V the sample is still in n-doped regime with $n=6.4x10^{9}$ cm^{-2} and μ = 61,000 cm^{2} V^{-1} s^{-1} .

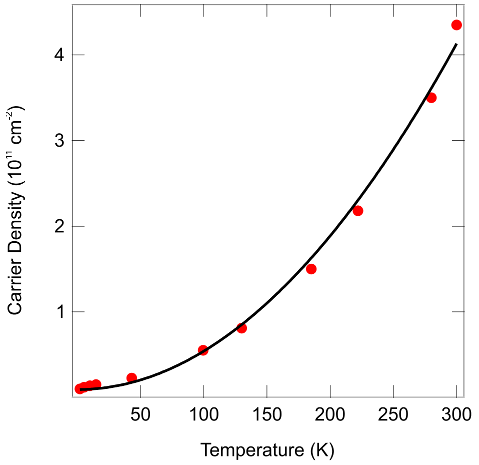

Supplementary Figure 6 - Charge homogeneity of chemical doping using two band model

From magnetotransport measurements, by looking at the minimum attainable carrier density n_{min} (Fig. 3) using electrostatic gating, we estimate the disorder strength of charge puddles to be $\Delta E_F = \pm\hbar v_F\sqrt{\pi n_{\mathrm{min}}} \approx \pm 9$ meV. For comparison, we again use disorder-induced temperature-dependent transport to estimate the average charge disorder in the doped SiC/G[2,3]. In detail, the model assumes that the spatial distribution of the potential V due to fluctuations follows a Gaussian probability distribution according to: $P(V) = \frac{1}{\sqrt{2\pi s^2}}\exp(-V^2/2s^2)$. The parameter s is a measure of the average strength of the potential fluctuations. With the assumption that SiC/G is close to charge neutrality, the electron and hole carrier densities can be expressed as: $n_{\mathrm{e}}(T) = n_{\mathrm{h}}(T) = \frac{8\pi}{(hv_{\mathrm{F}})^2}\left[\frac{s^2}{4} + \frac{(\pi k_{\mathrm{B}}T)^2}{12}\right]$. Fitting this equation to the temperature dependence of charge carrier concentration one can extract the disorder strength s. This has been done for 4 samples doped close to charge neutrality and on average, we observe s=7.7±1.2 meV (two standard deviations) as the average charge disorder with the lowest observed being s=7.1 meV. This corresponds well with the measured experimental value of ±9 meV.

Supplementary Note 2 - GIWAXs

Room temperature grazing-incidence wide angle x-ray scattering (GIWAXs) (Fig. 4), reveal a broad amorphous halo with a distinct diffraction peak at q=9.6 nm^{-1}, primarily from PMMA[4]. This is also evident from a reference GIWAXs measurement (Fig. 4b, c) using a dopant blend without F4TCNQ molecules (just PMMA on top of a PMMA spacer). Interestingly, the addition of F4TCNQ molecules enhanced the GIWAXs signal by a factor of 2. This could indicate that the molecules, embedded in the PMMA matrix, serve to stabilize the backbone of the polymer chains and/or follow the packing of PMMA itself. With a peak at q=9.6 nm^{-1}, the equivalent characteristic length scale for the F4TCNQ/PMMA system is 6.6 Å. If the molecules are 6.6 Å apart then the packing density corresponds to roughly 2 molecules per nm^2. Using Scherrer analysis one can extract the size of crystallites. The Scherrer equation is $\tau = \frac{K\lambda}{\beta\cos\theta}$, where τ is the mean size of ordered crystalline domains, K dimensionless shape factor (assuming spherical particle = 0.9), λ wave length of x-ray (1.162 Å), θ is the Bragg angle (0.15 degrees) and β the full width at half maximum (FWHM) of the diffraction peak for the sample with F4TCNQ (Fig. 4c). This yields a lower bound average coherence length of molecules of τ~2-3 nm, implying that molecular crystallites are only a few F4TCNQ molecules large at room temperature.

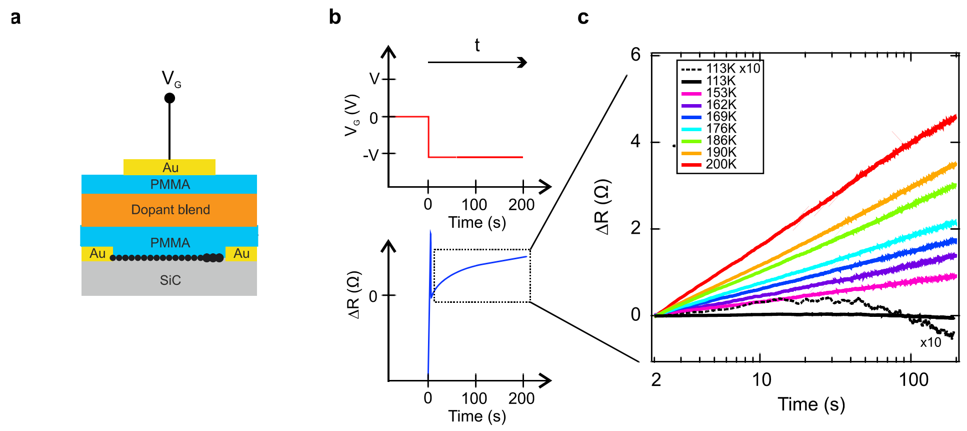

Supplementary Figure 7 - Redistribution of charges in the doping layer

To further investigate the charge redistribution we record the time-evolution of the sample resistance when a voltage step of V_G=-5 V (I_B=1 μA) is applied to the gate at different temperatures. (**a**) Schematic representation of chemically doped SiC/G with an electrostatic top gate. (**b**) At a fixed temperature and current bias, we apply a gate voltage which steps from V_G=0 V to V_G=-5 V, referenced to the drain electrode. When the top gate switches from V_G=0 V to negative voltage, the resistance of graphene increases due to reduced n-doping (graphene sample is n-doped $n \sim 10^{10}$ cm^{-2}). The initial response is fast (< 2 s) and the immediate change in resistance is on the order of 100 **Ω**. After the initial fast response there is a slower transient of increasing resistance. (**c**) The slower transients appear above ~100K and they tend to increase logarithmically with time. The relative change of resistance ΔR is referenced to the resistance value at time t=2 s, after all initial fast transients have settled down. Note that the relative change of the resistance ΔR during 200 s is <5 % of the total change in resistance immediately upon switching of the gate voltage. Each curve is an average of at least 10 gate switches. The response of the transients is symmetric with respect to gate polarity (not shown). The x10 magnified curve shows a downturn of resistance explained by temperature instability of our measurement over time, substantial for this precision measurements. At high temperature, the sample resistance evolves logarithmic in time ΔR ~log(t). As the sample is cooled down, we observe a slower response and at temperatures approaching ~100 K we observe that the time evolution essentially vanishes. Logarithmic time dependence of charge redistribution is typical for glass type of disorder in polymer-dopant blends.

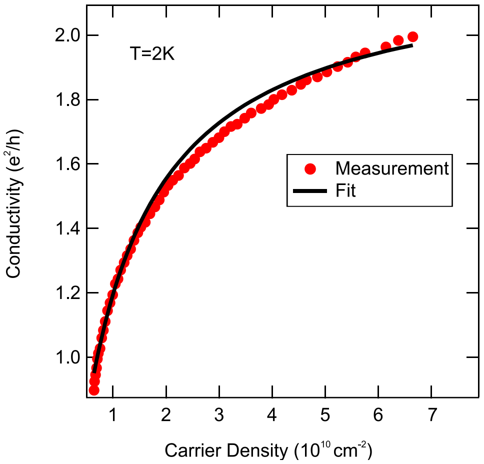

Supplementary Figure 8 - Transport in graphene with correlated disorder

Transport in chemically doped graphene (i.e. Fig. 3b) can be analyzed in the framework of 2D transport in graphene for correlated disorder[36]. The model describes how the conductivity in graphene depends on the charge carrier density in the presence of charged impurities with a degree of spatial correlation. The correlation length r_0 is bounded by the impurity density n_{i} as $r_0 \leq (\pi n_{\mathrm{i}})^{-1/2}$. The conductivity as a function of carrier density n, can be fitted to the equation $\sigma(n) = An[1 - a + Ba^2 n/n_i]^{-1}$. Where $A = \frac{e^2}{h}[2n_{\mathrm{i}} r_{\mathrm{s}}^2 G_1(r_{\mathrm{s}})]^{-1}$, $a = \pi n_{\mathrm{i}} r_0^2$ and $B = G_2(r_{\mathrm{s}})/(2G_1(r_{\mathrm{s}}))$. $r_s = e^2/(2\pi h v_{\mathrm{F}} \varepsilon_0 \kappa)$ is the effective fine structure constant for graphene in a dielectric environment with relative dielectric constant κ. $G_1(x) = \frac{\pi}{4} + 6x - 6\pi x^2 + 4x(6x^2 - 1)\frac{\mathrm{asech}(2x)}{\sqrt{1-4x^2}}$ and $G_2(x) = \frac{\pi}{16} + \frac{4x}{3} + 3\pi x^2 + 40x^3(1 - \pi x + \frac{4}{5}(5x^2 - 1)\frac{\mathrm{asech}(2x)}{\sqrt{1-4x^2}}$. The relevant parameters are r_0, n_{i} and r_s. n_{i} is in principle known from the estimates of the packing density of molecules and we use $3\text{x}10^{14}$ cm^{-2} (from transport, SIMS and GIWAXs, see main text). Fig. S8 shows that the model qualitatively fits our data and as fitting parameters we extract $r_0 \cong (\pi n_{\mathrm{i}})^{-1/2} = 2.3a_0$, and $r_s = 0.009725$. The correlation length is over twice the distance between two carbon atoms $a_0 = 1.42$ Å and the relative fine structure constant suggests an effective high-k environment. At very low carrier density, the conductivity σ(n) of chemically doped graphene displays the typical linear behavior for charged-impurity scattering, but appears to saturate at carrier densities exceeding the crossover density $n_c = (1 - a)n_i/(Ba^2) = 8.3\text{x}10^9$ cm^{-2}. This behavior is explained by scattering of graphene carriers on correlated charged impurities, which are the molecular dopants in our case.

Supporting references

1. Crank, J. *The mathematics of diffusion*. (Clarendon Press, 1975)

2. Huang, J. *et al.* Disorder induced Dirac-point physics in epitaxial graphene from temperature-dependent magneto-transport measurements. *Phys. Rev. B* **075407,** 6 (2015).

3. Li, Q., Hwang, E. H. & Das Sarma, S. Disorder-induced temperature-dependent transport in graphene: Puddles, impurities, activation, and diffusion. *Phys. Rev. B - Condens. Matter Mater. Phys.* **84,** (2011).

4. Ramanathan, T. *et al.* Functionalized graphene sheets for polymer nanocomposites. *Nat. Nanotechnol.* **3,** 327–31 (2008).
