# Supplementary material for: Uniform doping of graphene close to the Dirac point by polymer-assisted assembly of molecular dopants
Source: Nat Commun. 2018 Sep 27;9:3956. doi: 10.1038/s41467-018-06352-5 (PMC6160407; doi:10.1038/s41467-018-06352-5)
Supplement: Supplementary file 2 — Peer Review File [file 41467_2018_6352_MOESM2_ESM.pdf]

Editorial Note: this manuscript has been previously reviewed at another journal that is not operating a transparent peer review scheme. This document only contains reviewer comments and rebuttal letters for versions considered at Nature Communications.
